# Ophthalmia1Read before a meeting of the Wisconsin Society of Veterinary Graduates, March 14, 1900.

**Published:** 1900-05

**Authors:** G. Ed. Leech

**Affiliations:** Milwaukee, Wis.


### OPHTHALMIA.[1]

By G. Ed. Leech, M.D.C.,
MILWAUKEE, WIS.

(Concluded from page 214.)

*Sympathetic Ophthalmia* (in *Diseases of the Eye*, by Norris and Oliver). This case is reported by Deutchmann, and is of more than usual interest, because the man died of carcinoma of the stomach. The change found in the sympathizing eye, in the opinion of Deutchmann, must have been the result of a process that necessarily was limited to the visual apparatus from beginning to end.

A man, aged thirty years, had undergone an unsuccessful operation upon his right eye six months before; inflammation associated with great pain followed, and sight was ultimately lost. The patient when first seen presented the following conditions: In the right eye were phthisis bulbi and occlusion of the pupil; the cornea was clear, though there was a scar at the limbus marking the point where the incision was made at the time of the operation; slight ciliary injections were also present, and the eye was soft and extremely sensitive to touch or pressure. The other eye was sound so far as external appearance and vision were concerned. The ophthalmoscope, however, showed a red papilla with indistinct edges, the vessels being dilated and the surrounding retina cloudy. The patient refused absolutely to submit to an operation, so he was put through an inunction of sweating and iodide of potassium. It may be well to add that there was no evidence of any syphilis, though the man was very pale and had a cachectic appearance. He had no fever, and complained of nothing but a trouble in his stomach. The treatment with the above formula proved fruitless, the primarily affected eye grew less irritable, and gradually lost its sensitiveness to pressure, while the neuro-retinitis in the other eye increased, and vision

[1] Read before a meeting of the Wisconsin Society of Veterinary Graduates, March 14, 1900.

soon began to fail, followed by the appearance of fine opacities in the vitreous body. The patient was lost sight of, and when next seen the vision in his good eye was 1/xv, and there was present pronounced neuro-retinitis, opacities in the vitreous body, and beginning ciliary injection ; the other eye showed no further change. The cachectic condition of the patient seemed worse, and he presented the appearance of one afflicted with carcinoma. He still refused to allow an enucleation, so he was put under active treatment, with a view to bettering the condition of the other eye, the eye in which the sympathetic ophthalmia was present. Things continued to go from bad to worse, posterior synechia appeared, and the vitreous body became so cloudy that it was not possible to see the fundus. The vision sank to recognizing the movement of the hand, the general condition grew worse until there was no longer any doubt of the existence of carcinoma of the stomach. He died a few days later.

An autopsy was obtained and both orbits, the eyeballs, the optic nerves and the chiasms were secured for examination. It may be mentioned that at the autopsy the meninges showed nothing abnormal, the right eye was slightly atrophic, the optic nerve entrance decidedly spread out and infiltrated throughout with round cells. The trunk of the ciliary nerves at this point presented nothing abnormal ; sometimes here and there in the sheath a round cell would be noticed ; the tissue of the optic nerve itself was abundantly infiltrated with round cells, especially in the pial septa. The walls of the bloodvessels in the papilla were richly infiltrated with round cells. The retina, especially in the nerve-fibre layer, was markedly infiltrated, and at points was separated from the choroid by an exudate. The vessels of the retina participated to the same extent in the process. The retina in the vicinity of the ora serrata did not show much infiltration, but was simply atrophic. The choroid was atrophic very generally, and the pigment epithelium at points was detached and partly atrophied. Both the retinal infiltration and the choroidal infiltration were less pronounced near the ciliary region. The vitreous was detached anteriorly and posteriorly, and transformed into a fine fibrous tissue.

The sclera was slightly infiltrated at the posterior pole ; infiltration of the sclera as well as the sub-conjunctival tissue was noticed at the limbus of the cornea. The cornea was also infiltrated, and at its upper border was to be seen a scar, along the course of which cell-infiltration was very noticeable. The iris and ciliary body showed atrophy of the pigment elements, posterior synechiæ was present, the synechiæ consisting of nucleated fibrous tissue containing round cells, particles of pigment and endothelial cells, forming a tissue which completely closed the pupillary opening.

The anterior chamber contained an exudate that consisted of round cells and fibrin ; especially noteworthy was an anterior synechia unassociated with a corneal scar ; there was hardly more than a rudiment of the lens remaining.

As regards the condition of the second eye, the optic nerve where it passed into the eyeball was richly infiltrated with round cells, and this was particularly noticeable in the pial septa. The ciliary nerves at this point were intact, except that here and there a round cell could be noticed. The papillæ was very much swollen and infiltrated with round cells, and the sheaths of the central vessels participated in this process. The retina, especially in the fibre layer and as far forward as the ora serrata, was the seat of round-cell infiltrations, the infiltration showing even to some extent in the ora serrata. The choroid was richly infiltrated, the cells sometimes occurring in little groups or heaps, and sometimes being uniformly distributed throughout the tissue. The pigment epithelium was loosened. The round-cell infiltration of the choroid appeared to be less pronounced a short distance forward from the equator ; but beyond this, up to the ciliary body and in the latter, the cell infiltration was abundant. The sclera in general was intact, though the walls of the small bloodvessels penetrating the sclera were infiltrated.

The vitreous body was somewhat shrivelled and at some points was detached from the retina. Throughout the vitreous body were to be seen numerous cells, partly small round cells, partly large cells, with vacuoles and containing several nuclei. The posterior chamber contained coagulated fibrin mixed with round cells. The lens was intact, except that there was an abundant pigment deposit at the anterior capsule, and in addition to this an exudate that soldered the anterior capsule to the posterior surface of the lens. The ciliary body was densely infiltrated with round cells. The anterior chamber contained a fibrinous exudate rich in cells. The iris was infiltrated throughout with round cells, and the cornea showed a slight infiltration at the scleral border, as did also the sub-conjunctival tissue at this point. The external sheath of the optic nerve of the primarily affected eye all the way up to the chiasm was only moderately affected. The inner sheath, however, was considerably infiltrated with round cells, the infiltration being here and there quite dense and at other points more diffuse. The cell infiltration was pronounced in the pial septa. At the chiasm the inner sheath of the nerve was markedly infiltrated. The pia mater was infiltrated only in the immediate vicinity of the chiasm; otherwise it was perfectly normal.

The other optic nerve showed also pronounced round-cell infiltration of the inner sheath at the chiasm. This cell infiltration continued with variable intensity all the way down to the second eye.

Both orbits were normal, and there was here no suggestion of involvement on the part of nerves, muscles, or bloodvessels. The ciliary nerves wherever met with in the sections were perfectly normal. The brain and its membranes were normal. Micro-organisms were found in both eyes, and also in the optic nerves. The organisms had somewhat the appearance of gonococci, and in the primarily affected eye were observed to be most numerous in the ciliary body, iris, and choroid, especially in that part of the choroid near the papilla. They were also to be seen in the retina, in the papilla, and about the central vessels. In the optic nerve organisms were found in the nerve-trunk and in the walls of the smaller bloodvessels which pass through the inner sheath of the nerve. No organisms were found in the outer sheath of the nerve. The condition was the same in both optic nerves and in the chiasm. Some few organisms were found in the pia mater in the immediate vicinity of the chiasm. In the second eye microörganisms were found to be most abundant in the posterior part of the eye, just as was the case with the first eye. There were no organisms in the orbits.

It will be remembered that neuritis optica existed for some time in the second eye before there was disturbance of vision. Deutschmann thought that the neuritis was produced by the chemical and metabolic products that preceded the migration of the organism, and that it was only where the latter had traversed the optic nerves and passed into the second eye that the disease spread with rapidity and with its usual destructiveness. He regarded the optic nerves and chiasm as the route followed by the organisms, and believed that the pial sheath of the nerve and the nerve-trunk were the parts preferably attacked, and he concluded by stating his position again with respect to the pathogenesis of sympathetic ophthalmia—a position which is well known.

Such a case is of undoubted value, for if a general infection can be absolutely excluded we are not far from the solution of this problem. The observation, however, is an isolated one, and, while we cannot but appreciate its value, it will never be looked upon as conclusive so long as the experimental side of the question remains so one-sided.

It may be added that a general infection cannot possibly be excluded without a bacteriological examination of the blood and other organs, as such general infection, especially with streptococci, is not infrequent as a terminal event in various chronic diseases, including cancer of the stomach. The many negative results do not disprove the bacteriological origin of sympathetic ophthalmia, but before regarding such a theory as proven the specific organisms must be identified, and especially should this be the case with an infection like sympathetic ophthalmia, a disease the pathogenesis of which really does admit of more than one reasonable interpretation.

